# Surgery

**Published:** 1858-09

**Authors:** 


### SURGERY.

Excision of Bones and Joints.—[The following interesting statistical report of forty-three cases of excision of bones and joints, as occurring in the British Provincial hospitals during the year 1857, we extract from recent numbers of the *Medical Times and Gazette*. It will be seen that in fifteen cases the knee-joint was resected. Of these cases, there were six cures and four deaths; three cases, at time of report, were still under treatment, and doing well, while two were subjected to amputation three months after the primary operation. The results of this operation on the elbow-joint are far more favorable: thus, of seventeen cases, thirteen recovered, two died, in one amputation was performed, and one was still under treatment.]

*Case* 1.—The Bradford: Mr. Mead.—A fairly healthy lad, aged 19. Excision of the knee-joint on account of chronic synovial disease. Death from pyæmia on the thirteenth day. *Case* 2.—The Bradford: Mr. Mead.—A man, aged 47, in fair health. Excision of the scapula and head of humerus on account of malignant disease. There was considerable hemorrhage during the operation. Death from exhaustion two hours afterwards. *Case* 3.—The Leeds: Mr. Smith.—A girl, aged 20, of healthy appearance, for two years the subject of diseased elbow-joint. Several fistulous openings existed. Excision of the entire joint in the usual manner. Recovery. In this case cicatrization was complete within a month. *Case* 4.—The Liverpool: Mr. Bickersteth.—A strumous boy, aged 10, the subject of diseased elbow-joint. There were three or four sinuses around the joint, and the bones were extensively diseased. A free incision was performed, the H-incision being adopted. Recovered well. *Case* 5.—The Liverpool: Mr. Long.—A man, aged 25, in poor health. The subject of disease of the shoulder-joint. The joint was stiff, and several sinuses, both in front and behind, led into it. After some preparatory treatment, excision of the head of the humerus was performed. In the attempt to displace the head of the bone from the glenoid cavity, the shaft broke through at the insertion of the deltoid. The head of the humerus was found to be firmly anchylosed to the glenoid cavity, and some little trouble was required for its removal. The man did well. The fractured bone united firmly, and a fair amount of motion in the false joint was retained. Two sinuses still existed at the time that he was sent into the country for change of air. *Case* 6.—The Liverpool: Mr. Bickersteth.—A healthy lad, aged 12, the subject of disease of the elbow-joint. Excision of the joint by the H-shaped incision. Recovery, with a fair amount of motion. *Case* 7.—The Royal Berkshire Hospital.—A cachetic woman, aged 29, the subject of diseased shoulder-joint. Her strength failing, excision was determined on. The operation was performed in the usual manner; the head of the humerus was found extensively carious. The glenoid cavity was gouged. She suffered for some days from the shock of the operation, but afterwards did well. She was the subject of enlargement of the liver, and there was also a suspicion of lung disease. She left the hospital on April 24, the operation having been performed on March 12. The wound never entirely healed; death took place early in June. No autopsy. *Case* 8.—The Royal Berks.—A young woman, aged 23, in good health, but the subject of disease of the hip-joint of two years' standing. The disease had been acute only for three months, and was rapidly undermining her health. A single sinus in the thigh discharged profusely. After various measures of treatment, excision of the head of the femur was performed four months after her admission. The acetabulum was found much diseased and required free gouging. A finger could be passed through it into the cavity of the pelvis. She recovered well from the shock of the operation, but no reparative action was set up, and death took place on the fourth day. *Case* 9.—The Leeds: Mr. Teale.—A lad, aged 19. Excision of the elbow-joint, on account of chronic disease. The soft parts were much infiltrated and swollen, and healing consequently progressed slowly. Passive motion was commenced about a month after the operation. Recovery. *Case* 10.—Addenbrooke's: Mr. Humphry.—A healthy man, aged 31, for three years the subject of diseased ankle-joint. A complete excision was performed, the upper surface of the astragalus, and the lower end of the tibia, with both malleoli, being sawn away. No ill symptoms followed. Seven months afterwards the parts had healed, but the leg was still weak. *Case* 11.—Addenbrooke's: Mr Humphry.—A girl, aged 5, in delicate health, the subject of acute suppuration of the knee-joint, following old-standing disease of the patella. Excision of the joint. Diarrhœa was set up the next day, and continued to the time of death; death occurred on the twenty-second day, having been preceded by swelling of several of the joints. No autopsy was allowed. *Case* 12.—Addenbrooke's: Mr. Humphry.—A healthy laborer, aged 25, the subject of diseased knee-joint, following the kick of a horse. Excision of the joint. At first the case progressed very well, but subsequently abscesses formed about the bones. Seven months afterwards there were still some discharging sinuses, but the bones had firmly united. *Case* 13.—Addenbrooke's: Mr. Humphry.—A pale, unhealthy-looking man, aged 25, with disease of the knee-joint of three years' standing. Excision of the joint was performed. In the operation commencing ulceration of the cartilages was found, and numerous small polypoid growths from the synovial membrane; the latter were all removed. Erysipelas of the leg followed, and abscesses formed about the seat of the operation. Amputation through the thigh three months after the excision. Recovery. *Case* 14.—Addenbrooke's: Mr. Humphry.—A delicate lad, aged 10, for a year the subject of diseased knee-joint. There was an abscess in the ham, communicating with the joint. Excision of the joint. The edges of the wound separated, exposing a large suppurating surface. His health giving way, amputation through the thigh was performed three months after the excision. He recovered well. *Case* 15.—The Derby: Mr. Fearn.—A feeble lad, aged 18, the subject of diseased wrist. Excision of the diseased articular surfaces. Some sinuses still existed at the time he was made an out-patient. *Case* 16.—The Derby: Mr. Fearn.—A delicate girl, aged 16, the subject of disease of the elbow-joint. Excision of the joint. The disease extended, and amputation through the arm was performed a month afterwards. She recovered well. *Case* 17.—The Liverpool: Mr. Long.—A healthy-looking lad, aged 16, the subject of diseased elbow-joint, resulting from a blow a year ago. The actual cautery had been applied with marked benefit, but the disease still existed. The H-shaped incision was practiced, and the ends of the bone removed. He made an excellent recovery, and in less than three weeks could put his hand to his mouth; and in another three weeks could flex and extend the forearm quite freely. *Case* 18.—The Liverpool: Mr. Bickersteth.—A strumous lad, aged 18, the subject of old-standing disease of the elbow-joint. There were three or four sinuses which communicated with the joint. The H-incision was practiced, and the ends of the bone removed. The boy did well, and was discharged in much-improved health, and with fair motion in the elbow. *Case* 19.—The Liverpool: Mr. Bickersteth.—A feeble woman, aged 56, the subject of diseased elbow-joint. Excision of the articulation. The parts had nearly healed, when the patient's health failing, her friends removed her from the hospital. She shortly afterwards died. *Case* 20.—The North Staffordshire: Mr. Ball.—A girl, aged 12. Excision of the elbow-joint. Recovery, with good motion in the joint. *Case* 21.—The Staffordshire General: Dr. Masfen.—A healthy man, aged 37, the subject of diseased knee-joint. Excision of the joint, the patella being left. Death from exhaustion on the twenty-first day. *Case* 22.—Addenbrooke's: Mr. Humphry.—A girl, aged 18, pale, but in tolerable health, for seven years the subject of diseased knee-joint. The disease had been much aggravated by a sprain received three weeks before admission. There was much swelling, but no abscess had ever formed. Excision of the joint was performed. The patella, as well as the extremities of the two bones, was removed. The synovial membrane was found thickened; the cartilages were extensively ulcerated, and the ends of the bone were united by firm fibrous bands. Profuse suppuration followed the operation, but the wound gradually closed. Six months afterwards several sinuses still remained open. Under treatment. *Case* 23.—Addenbrooke's: Mr. Humphry.—A healthy woman, aged 27, was admitted with chronic disease of the knee-joint, consequent on a kick received nine years before. Excision of the joint was performed on October 25. The patella was removed, and the synovial membrane, which was much thickened, was dissected away; scarcely any suppuration followed, and the healing process was remarkably rapid.

She was up within six weeks of the operation, and was discharged at the end of the tenth week, the bones being firmly united. *Case* 24.—The Hull: Mr. Craven, Jr.—A strumous lad, aged 9, had suffered from disease of the right knee for two or three years. Excision of the joint was performed, the long semilunar incision being adopted; the patella was not removed. The case progressed well, and at the date of report anchylosis was almost complete. Under treatment. *Case* 25.—The Hull: Mr. Huntingdon.—A healthy man, aged 30, was admitted in consequence of an injury to his elbow-joint, inflicted by a circular saw. The olecranon process and the inner condyle of the humerus were cut through, but the vessels were uninjured. Mr. Huntingdon sawed off the other condyle, and removed also a further portion of the ulna and the articular surface of the radius. The arm was laid in a straight splint for three weeks, and afterwards placed on an angular one. The parts healed well, and a good amount of motion was secured in the new joint. *Case* 26.—The Dundee: Dr. Crockatt.—A strumous man, aged 20, was admitted, with disease of the right elbow-joint, consequent upon an injury. The joint was excised in the usual manner, and a good recovery, with a slight motion in the new joint, ensued. The articular surface of the ulna, and the lower end of the humerus, were extensively ulcerated. *Case* 27.—The Brighton: Mr. Lowdell.—A girl, aged 14, the subject of strumous disease of the elbow-joint, of eighteen months' duration. Excision was performed in the usual manner, and a good recovery followed. She left the hospital three months after the operation, having then a fair amount of motion in the joint, which was daily improving. *Case* 28.—The Glasgow.—A strumous boy, aged 10, the subject of chronic disease of the hip-joint. Excision of the head of the femur. Recovery. *Case* 29.—The Glasgow.—A man, aged 33, in good health. Primary excision of the ankle-joint, in consequence of a compound dislocation. Recovery. *Case* 30.—The Glasgow.—A strumous girl, aged 15, the subject of chronic disease of the elbow-joint. One ankle was also affected. Excision of the elbow was performed, but the child sank under hectic exhaustion at the end of the second month. *Case* 31.—The Glasgow.—A boy, aged 15, the subject of strumous disease of the elbow-joint. Excision. Recovery. *Case* 32.—The Glasgow.—A boy, aged 11, for nine months the subject of strumous disease of the elbow-joint. Excision. Recovery. *Case* 33.—The Glasgow.—A girl, aged 18, for four years the subject of strumous disease of the knee-joint. She was in much reduced health. Excision of the joint was performed in the usual way on July the 9th. A good recovery followed, and the consolidation of the parts was complete by the middle of December. Her general health had much improved, the anchylosis was perfect, and she could bear weight on the limb; the shortening was about an inch and a half. *Case* 34.—The Glasgow.—A boy, aged 10, for four years the subject of diseased knee-joint. Excision in the usual manner on December 28. At the date of report the case was doing well. *Case* 35.—The Glasgow.—A man, aged 33, was admitted with a compound dislocation of the elbow-joint, caused by a fall from a scaffold. The heads of the radius and ulna were broken, and were excised. The articular surface of the humerus was not interfered with. Recovery, with complete anchylosis, resulted. *Case* 36.—The Glasgow.—A man, aged 27, was admitted on November 3d, having had his elbow-joint opened by a circular saw; the olecranon and lower end of humerus had been injured, and were consequently removed. At the date of report the case was doing well. *Case* 37.—The Liverpool Royal: Mr. Bickersteth.—A delicate-looking boy, aged 11, the subject of diseased knee-joint. Excision was performed; the semilunar incision being adopted. The joint was found quite disorganized; the cartilages being almost wholly gone. The patella was not removed. A profuse discharge followed, and the boy was for some time in a critical condition. At the date of report there seemed good reason to hope the limb would be saved. *Case* 38.—The West Norfolk: Dr. Cotton.—A thin, spare woman, aged 45, was admitted with the intention of submitting to amputation of the thigh on account of diseased knee-joint. The disease had existed twenty-five years, and there was a large ulcer with fungoid granulations on the inner side of the joint. Excision of the joint was performed in the usual manner on September 28. She recovered well; and at the date of report was able to bear considerable weight on the limb. *Case* 39.—The West Norfolk: Dr. Cotton.—A delicate girl, aged 16, was admitted with chronic enlargement and contraction of the keee-joint: the disease was of nine months' duration. The joint was excised in the usual manner on September 28. For some time she appeared to be doing well, but she subsequently sank into a state of extreme anæmia, and so died in the beginning of the ninth week. Anasarca had been present during the last few weeks. The autopsy showed no disease of either lungs, heart, or kidneys, nor were there any evidences of pyæmia having existed. No osseous union had taken place between the extremities of the bones. *Case* 40.—The West Norfolk: Mr. Kendall.—A girl, aged 3 years, the subject of strumous disease of the knee-joint for more than twelve months. Excision of the joint was performed, and she made an excellent recovery. At the date of report she was able to bear weight on the limb. *Case* 41.—The Queen's Hospital, Birmingham: Mr. Sands Cox.—A healthy boy, aged 3, was admitted on account of a bony growth occupying the lower part of the scapula: it had been gradually increasing for two years. Mr. Cox excised the whole of the bone beneath its spine, by means of a chain-saw passed under it. No vessels required ligature, the hemorrhage being arrested by pressure. The tumor proved to be a flat-based exostosis. The healing was retarded by an attack of erysipelas. He was discharged well six weeks after the operation. *Case* 42.—The Leeds: Mr. Teale.—A boy, aged 9, the subject of diseased tarsus. The os calcis, and the greater part of the astragalus were excised. The suppuration was profuse for some weeks, but at length the parts healed well. At the time of discharge from the hospital, only a very small sinus remained. *Case* 43.—The Glasgow.—A boy, aged 8. Excision of the os calcis on account of strumous disease. Recovery.—*Med. Times and Gazette,* May 8th and June 5th, 1858.

